# Extranodal connective tissue invasion and the expression of desmosomal glycoprotein 1 in squamous cell carcinoma of the oesophagus.

**DOI:** 10.1038/bjc.1997.157

**Published:** 1997

**Authors:** S. Natsugoe, J. Mueller, F. Kijima, K. Aridome, M. Shimada, K. Shirao, C. Kusano, M. Baba, H. Yoshinaka, T. Fukumoto, T. Aikou

**Affiliations:** First Department of Surgery, Kagoshima University School of Medicine, Japan.

## Abstract

**Images:**


*British Journal of Cancer* (1997) **75**(6), 892–897
© 1997 Cancer Research Campaign

### Extranodal connective tissue invasion and the expression of desmosomal glycoprotein 1 in squamous cell carcinoma of the oesophagus

**S Natsugoe, J Mueller, F Kijima, K Aridome, M Shimada, K Shirao, C Kusano, M Baba, H Yoshinaka, T Fukumoto and T Aikou**

First Department of Surgery, Kagoshima University School of Medicine, Kagoshima 890, Japan

**Summary** We investigated extranodal connective tissue involvement (ECTI) in 39 patients with oesophageal carcinoma. Both the primary tumour and ECTI were immunohistochemically examined using the monoclonal antibody 32-2B for desmosomal glycoprotein 1 (DG1). Connective tissue carcinoma deposits were identified as cells within small lymph nodes, as lymphatic or venous vessel invasion or as widespread invasion beyond the capsule of metastatic lymph nodes. These histological findings were present in at least one area in 20 of 39 patients (51.3%). DG1 immunostaining intensity by tumour was graded as DG1(++), DG1(+) or DG1(–). DG1(+) or DG1(–) primary tumours demonstrated lymph node metastases and ECTI more frequently than DG1(++) tumours ($P$<0.05). Among 17 patients in whom DG1 immunohistochemistry was performed on ECTI, there were three DG1(++), five DG1(+) and nine DG1(–) patients. The DG1 expression of ECTI was equal to or less intense than the primary tumour. These results indicate that reduction or loss of DG1 expression may promote ECTI and lymph node metastases. One should be aware of the potential for ECTI in oesophageal carcinomas. In the future, adjuvant therapy may be advisable for some oesophageal carcinomas based on the phenotype of individual cancer cells, including expression of DG1.

**Keywords:** oesophageal cancer; extranodal connective tissue invasion; lymph node metastasis; desmosomal protein; cell adhesion molecule

The prognosis of a patient with carcinoma of the oesophagus is poor compared with that of patients with carcinomas of other areas of the digestive tract. Although the prognosis of patients with oesophageal carcinoma has been improved by the use of extended lymph node dissections (Siewert and Roder, 1992; Akiyama et al, 1994, Baba et al, 1994), the incidence of recurrence is still high (Sugimachi et al, 1983; Chan et al, 1986; Natsugoe et al, 1994). Even if metastatic lymph nodes are removed surgically, some patients will have locoregional recurrence. We have previously reported (Yoshinaka et al, 1991) that, when the resected lymph nodes were examined in detail, perinodal invasion (cancer cells in the connective tissue within a 2-mm zone surrounding the lymph node) was observed in 29 of 73 patients (40%) and that the prognosis of these patients was poorer than that of patients without this finding.

In the present study, we histologically examined extranodal connective tissue involvement (ECTI) by oesophageal carcinoma which we defined as tumour cells present in connective tissue at a distance of more than 2 mm from the lymph node. Immunohistochemical studies, using the monoclonal antibody 32-2B to desmosomal glycoprotein 1 (DG1) were performed. Desmosomes are transmembrane structures with elements composed of various glycoproteins, notably desmoglein and desmocollin, both of which have recently been identified as members of the large family of cadherins (Goodwin et al, 1990; Wheeler et al, 1991). The desmosomal glycoprotein is a transmembrane molecule present in a wide variety of epithelia. The 32-2B antibody reacts reliably with epithelia and epithelial tumours in fixed, paraffin-embedded sections (Vilela et al, 1987). Recently, Vilela et al (1995) reported that this antibody recognized the cytoplasmic domains of both desmoglein 1 and desmoglein 3. The epidermal isoforms desmoglein 1 and desmoglein 3 are restricted to certain specialized, mostly stratified squamous, epithelia (Schäfer et al, 1994). There have been some studies of DG1 expression using the antibody 32-2B against transitional cell carcinoma of the bladder (Conn et al, 1990), oral squamous cell carcinoma (Imai et al, 1991; Harada et al, 1992) and oesophageal squamous cell carcinoma (Natsugoe et al, 1995*a*).

The purpose of this study was to examine ECTI in the oesophageal carcinoma and to compare it with the immunohistochemical expression of DG1.

### PATIENTS AND METHODS

### Patients

Thirty-nine patients with carcinoma of the oesophagus who had undergone treatment at the First Department of Surgery, Kagoshima University Hospital, were enrolled in this study for the period April 1992 to December 1993. Twenty-seven patients underwent oesophagectomy combined with extensive dissection of cervical, mediastinal and abdominal lymph nodes. Twelve patients did not have a cervical lymph node dissection because of advanced age or illness. The ages of the patients ranged from 45 to 75 years (mean 62.9 years); there were 38 men and one woman. None of the patients received radiation therapy or chemotherapy before surgical treatment.

*Received 25 January 1996*
*Revised 30 August 1996*
*Accepted 13 September 1996*

*Correspondence to:* S Natsugoe, First Department of Surgery, Kagoshima University School of Medicine, 8–35–1 Sakuragaoka, Kagoshima 890, Japan

**Figure 1** (**A**) Cancer metastasis to a very small lymph node less than 2 mm in diameter (arrow, cancer cells) (×40). (**B**) Lymphatic invasion into the connective tissues (arrow, cancer cells; L, lymphatic vessel) (×100). (**C**) Venous invasion into the connective tissues (arrow, cancer cells; V, vein) (× 100). (**D**) Widespread invasion into connective tissues beyond the capsule of the metastatic lymph node (arrow, cancer cells) (×100)

Post-operative radiation therapy or chemotherapy was given to 12 patients. All patients were followed up after discharge as follows: a radiographic examination was done every 1–3 months, computerized tomography every 3–6 months and ultrasonography every 6 months. Bronchoscopic and endoscopic examination was performed when necessary. We classified the types of carcinoma recurrence as: (1) locoregional recurrence including the neighbouring oesophageal bed and regional lymph nodes; (2) haematogenous recurrence; and (3) mixed recurrence. Follow-up data after surgery were available for all patients with a median follow-up period of 10 months (range 1–24 months).

Based on the TNM classification of the International Union Against Cancer (1987), the 39 patients were divided into ten patients with T1 tumours, four patients with T2 tumours, 15 patients with T3 tumours and ten patients with T4 tumours. One tumour was located in the upper one-third of the oesophagus, 24 tumours in the middle one-third and 14 tumours in the lower one-third. Pathologically, all the tumours were squamous cell carcinoma (11 well-differentiated, 19 moderately differentiated and nine poorly differentiated).

Lymph node metastases were present in 26 of 39 of the cases (66.7%). All of the M1 category tumours were due to distant lymph node metastases. The number of resected nodes per patient ranged from 16 to 107, with a median of 60 and the number of lymph node metastases ranged from 0 to 54, with a mean of eight.

**Table 1** Relationship between ECTI and TNM pathological classification

| | ECTI- absent ($n = 19$) | ECTI present ($n = 20$) | $P$-value |
|---|---|---|---|
| pT | | | < 0.05 |
| T1 | 8 | 2 | |
| T2 | 3 | 1 | |
| T3 | 5 | 10 | |
| T4 | 3 | 7 | |
| pN | | | < 0.01 |
| N0 | 11 | 2 | |
| N1 | 8 | 18 | |
| pM | | | NS |
| M0 | 16 | 11 | |
| M1 | 3 | 9 | |
| Stage | | | < 0.05 |
| I | 7 | 1 | |
| IIA | 3 | 1 | |
| IIB | 3 | 1 | |
| III | 3 | 8 | |
| IV | 3 | 9 | |

NS, not significant.

© Cancer Research Campaign 1997

**Figure 2** Immunoreactive DG1 expression in normal oesophageal epithelial cells. Normal epithelial cells strongly expressed DG1

### Preparation of histological sections

Before this study, as part of the routine histological examination following surgery, lymph nodes were macroscopically isolated individually from the resection specimen along with approximately 2 mm of surrounding tissue. These lymph nodes were labelled according to the areas that corresponded to the areas designated by the Japanese Society for Esophageal Disease (JSED) (1992). In this study, after the lymph nodes had been macroscopically isolated, the residual connective tissue around the excised lymph nodes was separated and classified in the same fashion as the lymph nodes for histological examination. Very small lymph nodes less than 2 mm in diameter, which could not be macroscopically separated from surrounding connective tissues, were also included in this study. Areas of connective tissue surrounding primary tumours which macroscopically invaded the para-oesophageal tissues were excluded from this study.

The tissues for study were fixed in 10% formalin and embedded in paraffin. Serial 4-μm thick sections of the connective tissue were prepared at three different levels, divided into three nearly equal parts: upper, middle and lower. Tissue sections were mounted on glass slides and stained with haematoxylin and eosin (HE). A total of 1881 histological sections were prepared for microscopic examination.

### Immunohistochemical staining

The tissue sections were deparaffinized with xylene, dehydrated with 98% ethanol and stained using an avidin–biotin–immunoperoxidase technique (ABC method). To block endogenous peroxidase activity, the sections were immersed in a 0.3% hydrogen peroxidase–methanol solution for 30 min and then washed with phosphate-buffered saline (PBS; pH 7.2) three times for 5 min each.

The sections were first incubated with mouse serum diluted 100-fold with PBS for 30 min at room temperature. After washing with PBS, the sections were incubated at 4°C overnight with the monoclonal antibody 32–2B for desmosomal glycoprotein 1 (Sanbio, USA) diluted 20-fold in 1% bovine serum albumin in PBS. After washing with PBS, the specimens were incubated with biotinylated rabbit anti-mouse IgG (Vector Laboratories, USA) for 30 min at room temperature. After washing with PBS, avidin-conjugated peroxidase (Vector Laboratories, USA) was added, and the incubation was continued for 60 min. The chromogen was developed with 0.01% diaminobenzidine, and the sections were counterstained with Mayer's haematoxylin and then coverslipped using glycerol gelatin.

### Immunohistochemical evaluation

The intensity and pattern of DG1 staining in the carcinoma cells of both the primary tumour and connective tissues were interpreted as follows: DG1(++), cells with strong staining (i.e. the same as that of normal epithelium); DG1(+), cells with weaker staining intensity than that of normal epithelium or some cells with the same as that of normal epithelium, but others with weaker staining; and DG1(–), cells without staining.

### Statistical evaluation

The data obtained were statistically compared using a chi-square test, with a *P*-value of less than 0.05 considered to be significant.

### Clinicopathological data vs ECTI

Histological examination of the connective tissues revealed tumour in the following morphological patterns: (1) very small lymph node less than 2 mm in diameter which could not be separated macroscopically from surrounding connective tissues; (2) lymphatic invasion; (3) venous invasion; and (4) widespread invasion of the connective tissues with cancer cells infiltrating beyond the capsule of metastatic lymph nodes (Figure 1). At least one area of ECTI was present in 20 of 39 patients (51.3%) and 43 regional connective tissue samples according to classification of code number by JSED (1992).

The relationship between ECTI and TNM stage is summarized in Table 1. Of the 25 patients with T3 or T4 tumours, ECTI was present in 17. However, ECTI was also found in two of ten patients with T1 tumours. Two of thirteen patients without lymph node metastases (N0) had ECTI. ECTI was present in five of eight patients who had between one and four positive nodes, in four of six patients who had five and nine positive nodes and in 9 of 12 patients who had ten or more positive nodes.

### DG1 expression vs clinicopathological findings

Normal squamous epithelial cells strongly expressed DG1 protein at cell–cell boundaries (Figure 2). Of the 39 primary tumours examined, 14 (35.9%) tumours were strongly positive for desmoglein 1 [DG1(++)], 15 (38.5%) tumours were weakly positive for desmoglein 1 [DG1(+)], and ten (25.6%) were negative for desmoglein 1 [DG1(–)] (Figure 3). Lymph node metastases were observed in five (35.7%) of the DG1(++) tumours, 12 (80.0%) of the DG1(+) tumours and nine (90.0%) of the desmoglein 1 negative tumours. The difference between the percentage of DG1(++) tumours and weak or negative tumours was statistically significant ($P < 0.05$) (Table 2) with respect to metastases.

A similar relationship between the ECTI and DG1 expression in primary tumour was seen, with the ECTI rates in DG1(++), DG1(+) and DG1(–) being 21.4%, (3/14) 60% (9/15) and 80% (8/10) respectively. There were statistically significant differences between the DG1(++) and DG1(+) groups ($P < 0.05$), and between

© Cancer Research Campaign 1997

**Figure 3** Immunohistochemical expression of DG1 in a primary oesophageal tumour. (**A**) DG1(++) tumour. All of the tumour cells strongly express DG1. (**B**) DG1(+) tumour. DG1 expression in all tumour cells was weaker than in normal epithelial cells. (**C**) DG1(+) tumour. Some tumour cells expressed DG1 strongly, but others weakly. (**D**) DG1(–) tumour. No DG1 expression was seen in the tumour cells

**Table 2** DG1 expression of primary tumour with or without lymph node metastasis

| | DG1 expression (%) | | | |
|---|---|---|---|---|
| | (++) | (+) | (–) | Total |
| LN negative | 9 (64.3) | 3 (20.0) | 1 (10.0) | 13 (33.3) |
| LN positive | 5 (35.7) | 12 (80.0) | 9 (90.0) | 26 (66.7) |
| | (++) vs (+): * | | (++) vs (–): * | |

*$P < 0.05$.

**Table 3** DG1 expression of primary tumour with or without ECTI

| | DG1 expression (%) | | | |
|---|---|---|---|---|
| | (++) | (+) | (–) | Total |
| ECTI negative | 11 (78.6) | 6 (40.0) | 2 (20.0) | 19 (48.7) |
| ECTI positive | 3 (21.4) | 9 (60.0) | 8 (80.0) | 20 (51.3) |
| | (++) vs (+): * | | (++) vs (–): ** | |

*$P < 0.05$. **$P < 0.01$.

the DG1(++) and DG1(–) groups ($P < 0.01$) (Table 3) with respect to ECTI.

The immunohistochemical expression of DG1 in the tumour cells of the foci of ECTI was studied in the same manner as in the primary tumour. However, the small foci of cancer cells disappeared in the additional sections prepared for immunohistochemistry in 8 of 43 lesions in the 20 patients with ECTI seen on HE staining. The remaining 35 lesions of 17 patients were evaluated immunohistochemically. There was no discrepancy among multiple foci of ECTI in terms of DG1 expression from the same patients. Of these 17 patients, the numbers of lesions with DG1(++), DG1(+) and DG1(–) tumour cells were three, five and nine respectively (Figure 4). When DG1 expression between the primary tumours and ECTI was compared, all of the three primary tumours with DG1(++) and ECTI also had DG1(++) in their ECTI foci. In five of seven primary tumours with DG1(+) and ECTI, the ECTI also had DG1(+); and all seven primary tumours with DG1(–) and ECTI also had ECTI foci that were DG1(–) (Table 4). No ECTI focus was observed in which the DG1(++) expression of the primary tumour changed to DG1(+) or DG1(–) in the ECTI, or in which DG1(+) or DG1(–) expression in the primary tumour changed to DG1(++) in the ECTI focus (Table 4).

Clinically, 2 of the 35 patients in this series died of post-operative complications, and 17 patients had recurrent disease after

© Cancer Research Campaign 1997

**Figure 4** Immunohistochemical expression of DG1 in the ECTI (**A**) DG1(++) tumour. (**B**) DG1(–) tumour

**Table 4** Relationship between DG1 expression and recurrence

| Case no. | DG1 expression in primary tumour | DG1 expression in ECTI | Mode of recurrence |
|---|---|---|---|
| 1 | – | – | L |
| 2 | – | – | L |
| 3 | – | – | L |
| 4 | – | – | M |
| 5 | – | – | H |
| 6 | – | – | P |
| 7 | – | – | |
| 8 | + | – | L |
| 9 | + | – | |
| 10 | + | + | L |
| 11 | + | + | L |
| 12 | + | + | L |
| 13 | + | + | M |
| 14 | + | + | |
| 15 | ++ | ++ | P |
| 16 | ++ | ++ | |
| 17 | ++ | ++ | |

L, locoregional recurrence; H, haematogenous recurrence; M, mixed recurrence; P, post-operative hospital death.

surgery. Recurrent legions were locoregional in nine patients, haematogeneous in four patients and mixed in four patients. Among the patients in whom DG1 expression of ECTI was immunohistochemically studied, recurrence was found in 10 of 15 patients, excluding the two patients who died of post-operative complications. These ten patients had either DG1(–) or DG1(+) expression in the tumour cells of both the primary lesion and the ECTI foci. Concerning the mode of recurrence, locoregional recurrence was the predominant pattern in seven of these ten patients (Table 4).

### DISCUSSION

Lymph node metastasis is one of the most important prognostic factors for determining the outcome of patients with oesophageal cancer. Even if a complete lymph node dissection is performed and no lymph node metastases are found, some patients still suffer from recurrent disease. Occult metastases have been found in detailed histological examinations by means of additional tissue sections. The incidence of occult lymph node metastases have been reported to range from 2% to 33% (Kingsley et al, 1985; International Breast Cancer Study Group, 1990; Natsugoe et al, 1991). Although many reports concerned with lymph node metastases have been published, there are few reports on ECTI. One must also pay special attention to connective tissues, because cancer cells move through the lymphatics and veins of the connective tissues. Fifty-one per cent of the 39 patients in this study had tumour involving the para-oesophageal connective tissues. Almost all of the lesions consisted of small numbers of cancer cells. These lesions could be detected neither macroscopically nor by routine histological examination.

Desmosomes act as intercellular junctions which provide sites of strong adhesion between epithelial cells. According to electron microscopic study, a decrease in the number of desmosomes in invasive carcinoma may contribute to reduction in cell adhesion and to metastatic potential (Arloy et al, 1981). We have also previously reported a decrease in the number of desmosomes in oesophageal cancer cells compared with normal epithelium, as shown by electron microscopy (Aikou et al, 1993). However, electron microscopic studies are necessarily based on small samples of tissue. The use of immunohistochemistry enables the study of much larger areas of a tumour and allows a more accurate impression of the overall distribution and number of desmosomes.

When the relationship between DG1 expression of the primary tumour and the presence of lymph node metastases was observed, lymph node metastases were found to occur more frequently in tumours with weak or negative expression of DG1 than in tumours which strongly expressed DG1. As we also saw in our study, Harada et al (1992) reported that the immunohistochemical DG1 expression of the tumour in metastatic lymph nodes of oral squamous cell carcinoma was as weak as that of the primary tumour. A tumour with reduction or loss of DG1 expression may be more likely to metastasize to lymph nodes.

Furthermore, in our study, the frequency of ECTI was lower in tumours that strongly expressed DG1. On the other hand, ECTI frequently occurred in tumours in which DG1 expression was reduced or lost. DG1 expression in the tumour cells of the ECTI foci was unchanged or reduced in all patients comapred with the DG1 expression of the corresponding primary tumour. These findings suggest that partial or complete loss of DG1 expression by

© Cancer Research Campaign 1997

cancer cells may promote ECTI as well as lymph node metastases. In other words, once the desmosomal attachment structure becomes impaired, the risk of metastases increases. However, three patients had tumours with strong DG1 expression in both the primary tumour and in the ECTI foci. It is possible that, in these tumours, the DG1 recognized by the 32–2B antibody is non-functional.

The frequency of recurrence in patients with weak or negative expression of DG1 tumours was high despite the short follow-up period. The mode of recurrence of these patients was most commonly locoregional. These results fit with the clinical progression of these tumours as these patients also had both lymph node metastases and ECTI.

The persistence of tumour cells in the form of residual deposits of ECTI not removed by surgery treatment may be responsible for these recurrences. This would be an argument in favour of the use of various forms of adjuvant therapy (Kelsen et al, 1990 Orringer et al, 1990). A targeted drug delivery system might be a useful means for obtaining high concentrations of anti-cancer agents in occult lymph node metastases or ECTI (Hagiwara et al, 1988; Natsugoe et al, 1995*b*).

In this study, we demonstrated a high frequency of ECTI in oesophageal cancer which appears to be a factor in the tumour recurrence. The relative lack of expression of DG1 may identify those oesophageal tumours which are more likely to metastasize in the form of nodal metastases or ECTI. In the future, adjuvant therapy may be an important tool in eradicating tumour cells in the form of occulat nodal metastases and ECTI that cannot be removed surgically.

### ACKNOWLEDGEMENT

This study was supported in part by grants-in-aid for scientific research from the Ministry of Education, Science and Culture, Japan.

### REFERENCES

Aikou T, Natsugoe S, Shimada M and Shimazu H (1993) A comparative study on fine structure of inflammatory and neoplastic dysplasia of the esophagus. *Med Electron Microsc* **26**: 89–97

Akiyama H, Tsurumaru M, Udagawa H and Kajiyama Y (1994) Radical lymph node dissection for cancer of the thoracic esophagus. *Ann Surg* **220**: 364–373

Alroy J, Pauli Bu and Weinstein RS. (1981). Correlation between numbers of desmosomes and the aggressiveness of transitional cell carcinoma in human urinary bladder. *Cancer*, **47**: 104–112

Baba M, Aikou T, Yoshinaka H, Natsugoe S, Fukumoto T, Shimazu H and Akazawa K (1994). Long-term results of subtotal esophagectomy with three-field lymphadenectomy for carcinoma of the thoracic esophagus. *Ann Surg*, **219**: 310–316

Chan KW, Chan EYW, and Chan CW. (1986). Carcinoma of the esophagus. An autopsy study of 231 cases. *Pathology* **18**: 400–405

Conn IG, Vilela J, Garrod DR, Crocker J and Wallace MA. (1990). Immunohistochemical staining with monoclonal antibody 32–2b to desmosomal glycoprotein 1. Its role in the histological assessment of urothelial carcinomas. *Br J Urol* **65**: 176–180

Goodwin L, Hill J, Raynor K, Paszi L, Manabe M and Cowin P. (1990). Desmoglein shows extensive homology to the cadherin family of cell adhesion molecules. *Biochems Biophy Res Commun* **173**: 1224–1230

Hagiwara A, Takahashi T, Ueda T, Iwamoto A, Yamashita H and Maeda T (1988) Enhanced therapeutic efficacy on lymph node metastasis by the use of Peplomycin adsorbed on small activated carbon particles. *Anti-cancer Res* **8**: 287–290

Harada T, Shinohara M, Nakamura S, Shimada M and Oka M. (1992). Immunohistochemical detection of desmosomes in oral squamous cell carcinomas: correlation with differentiation, mode of invasion, and metastatic potential. *Int Oral Maxillofac Surg* **21**: 346–349

Imai K, Kumagai S, Nakagawa K, Yamamoto E and Kawahara E. (1991). A pathological evaluation of intercellular adhesion by use of desmosome for squamous cell carcinoma of the oral cavity. *Jpn J Clin* **37**: 1779–1784

International Union Against Cancer. Hermanek P and Sobin LH (1987) *TNM-Classification of Malignant Tumors* 4th edn. Springer-Verlag: Berlin pp. 40–42

International Breast Cancer Study Group (1990) Prognostic importance of occult axillary lymph node metastases from breast cancers. *Lancet* **335**: 1565–1568

Japanese Society for Esophageal Disease (1992) *Guidelines for the Clinica and Pathologic Studies on Carcinoma of the Esophagus* 8th edn. Kanehara: Tokyo

Kelsen DP, Minsky B, Smith M, Beitler J, Niedzwiecki D, Chapman D, Bains M, Burt M, Heelan R and Hilaris B (1990) Preoperative therapy foe esophageal cancer: a randomized comparison of chemotherapy versus radiation therapy. *J Clin Oncol* **8**: 1352–1361

Kingsley WB, Peters GN and Cheek JH (1985) What constitutes adequate study of axillary lymph nodes in breast cancer? *Ann Surg* **201**: 311–314

Natsugoe S, Aikou T and Shimazu H (1991) A detailed histological study on occult metastasis of the lymph nodes. *Jpn J Surg* **21**: 528–532

Natsugoe S, Shimazu H, Yoshinaka H, Baba M, Fukumoto T and Aikou T (1994) Recurrence of thoracic esophageal cancer after three-field lymphadenectomy. In *Recent Advances in Disease of the Esophagus*, Nabeya K, Hanaoka T and Nogami H (eds), pp. 759–799. Springer-verlag: Tokyo.

Natsugoe S, Sagara M, Shimada M, Kumanohoso T, Tokuda K, Wakamatsu D, Tezuka Y, Kusano C, Yoshinaka H, Baba M, Fukumoto T and Aikou T (1995*a*) Expression of desmoglein I cell adhesion molecule in primary tumors and metastatic lymph nodes of esophageal cancer. *Int J Oncol* **6**: 345–348

Natsugoe S, Shimada M, Kumanohoso T, Tokuda K, Baba M, Yoshinaka H, Fukumoto T, Nakamura K, Yamada K, Nakashima T and Aikou T (1995*b*) Enhanced efficacy of bleomycin adsorbed on silica particles against lymph node metastasis in patients with esophageal cancer: a pilot study. *Surgery* **117**: 636–641

Orringer MB, Forastiere AA, Pera-Tamayo C, Urba S, Takasugi BJ and Bromberg J (1990) Chemotherapy and radiation therapy before transhiatal esophagectomy for esophageal carcinoma. *Ann Thorac Surg* **49**: 348–355

Schafer S, Koch PJ and Franke WW (1994) Identification of the ubiquitous human desmoglein, Dsg2, and the expression catalogue of the desmoglein subfamily of desmosomal cadherins. *Exp Cell Res* **211**: 391–399.

Siewert JR and Roder JD (1992) Lymphadenectomy in esophageal cancer surgery. *Dis Esoph* **2**: 91–97

Sugimachi K, Inokuchi K, Kuwano H, Kai H and Okamura Y (1983) Patients of recurrence after curative resection for carcinoma of the thoracic part of the esophagus. *Surg Gynecol Obstet* **157**: 537–540

Vilela MJ, Parrish EP, Wright DH and Garrod DR (1987) Monoclonal antibody to desmosomal glycoprotein 1 – a new epithelial marker for diagnostic pathology. *J Pathol* **153**: 365–375

Vilela MJ, Hashimoto T Nishikawa T, North AJ and Garrod D (1995) A simple epithelial line (MDCK) shows heterogeneity of desmoglein isoforms, one resembling pemphigus vulgaris antigen. *J Cell Sci* **108**: 1743–1750

Wheeler GN, Rutman AJ, Pidsley SC, Watt FM, Rees DA, Buxton RS and Magee AI (1991) Desmosomal glycoprotein DGI, a component of intercellular junctions, is related to the cadherin family of cell adhesion molecules. *Proc Natl Acad Sci USA* **88**: 4796–4800

Yoshinaka H, Shimazu H, Natsugoe S, Haraguchi Y, Shimada M, Baba M and Fukumoto T (1992) Histopathological features of the lymph node metastases in patients with thoracic esophageal cancer. *Nippon Gekagakkai Zassi* **93**: 1289–1296

© Cancer Research Campaign 1997


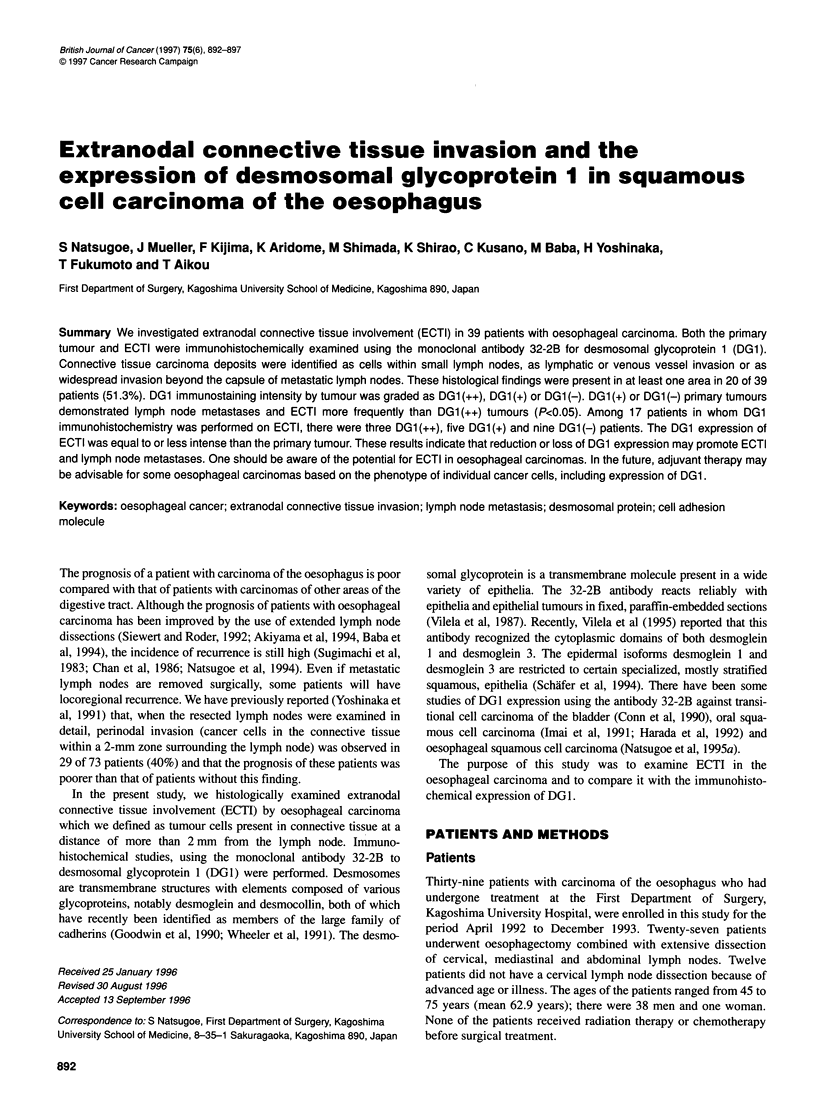

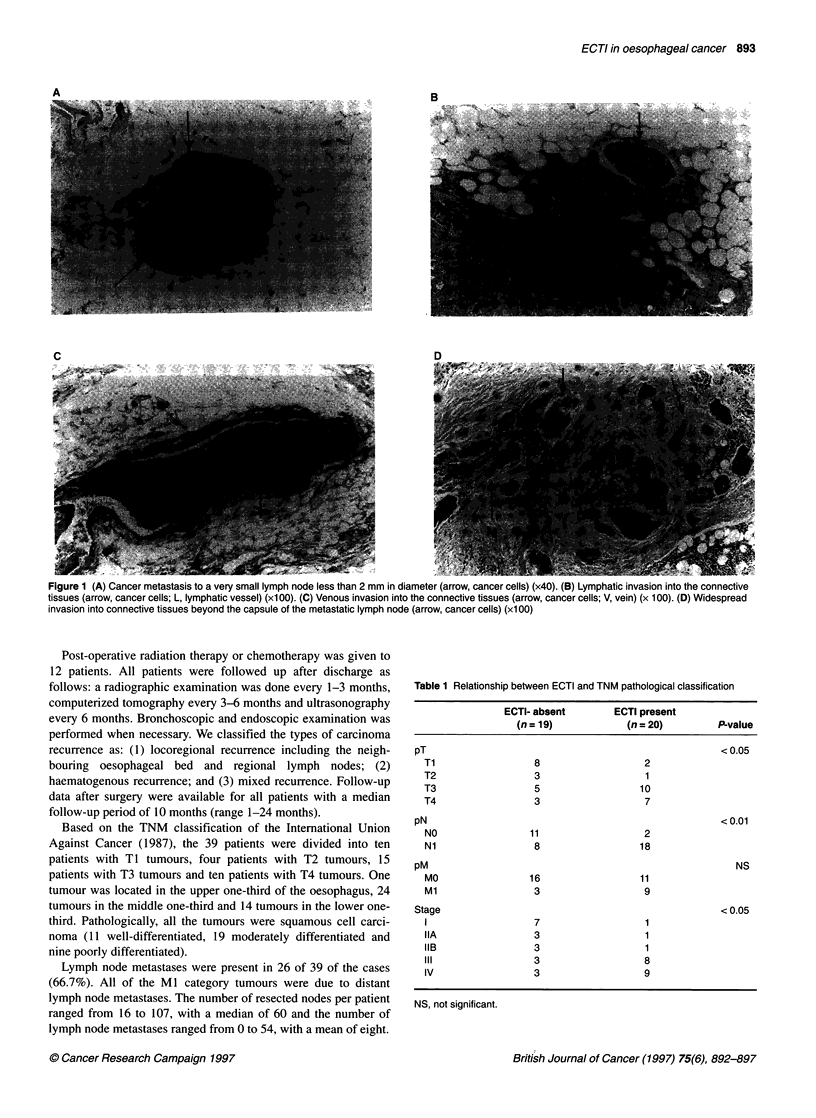

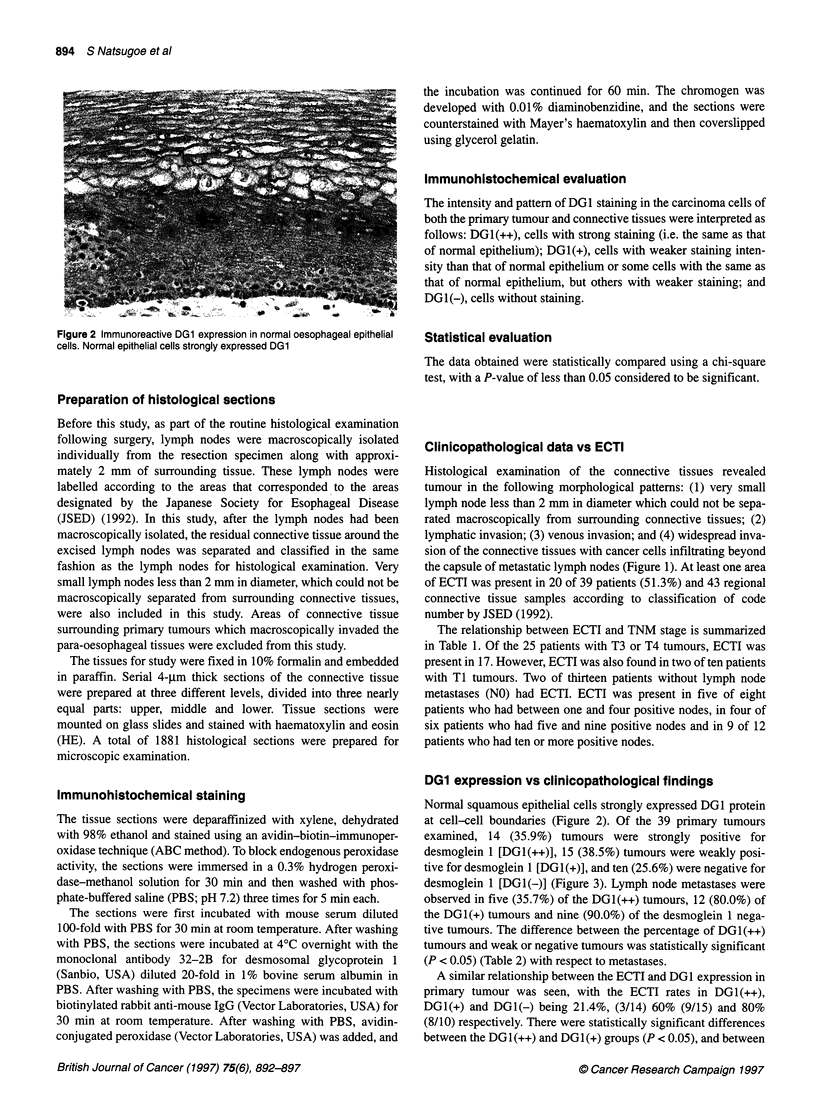

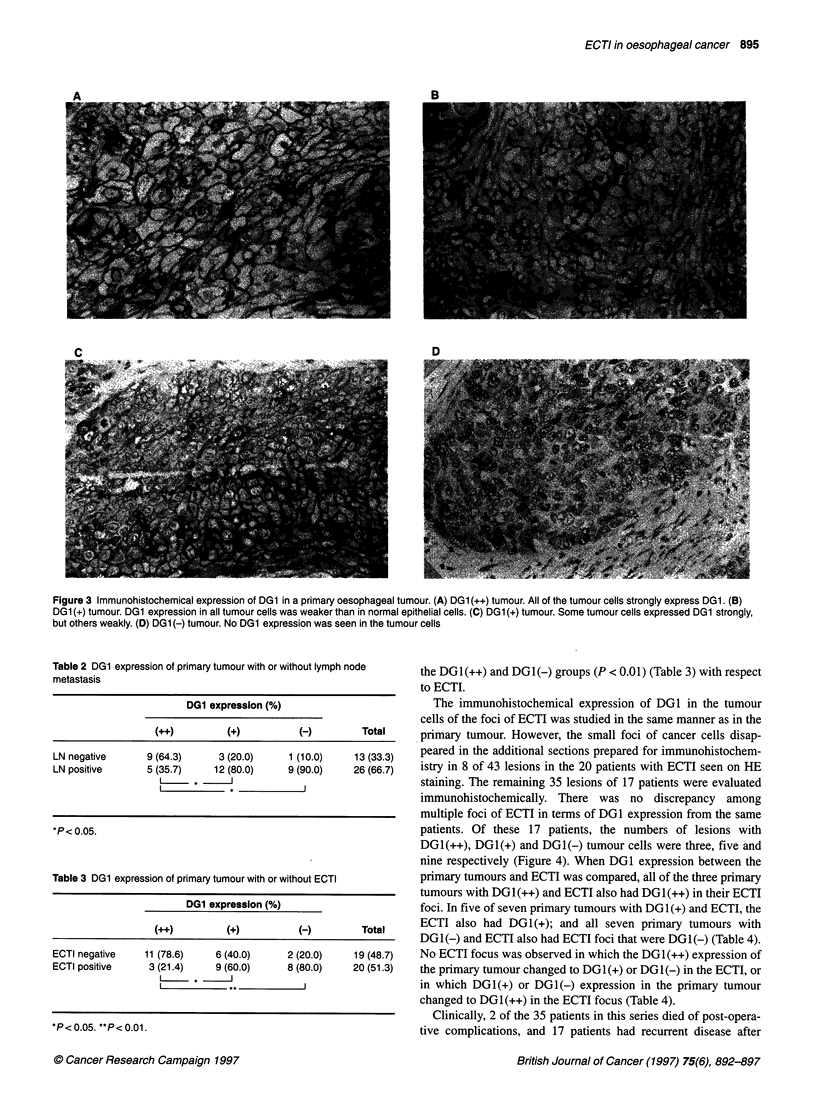

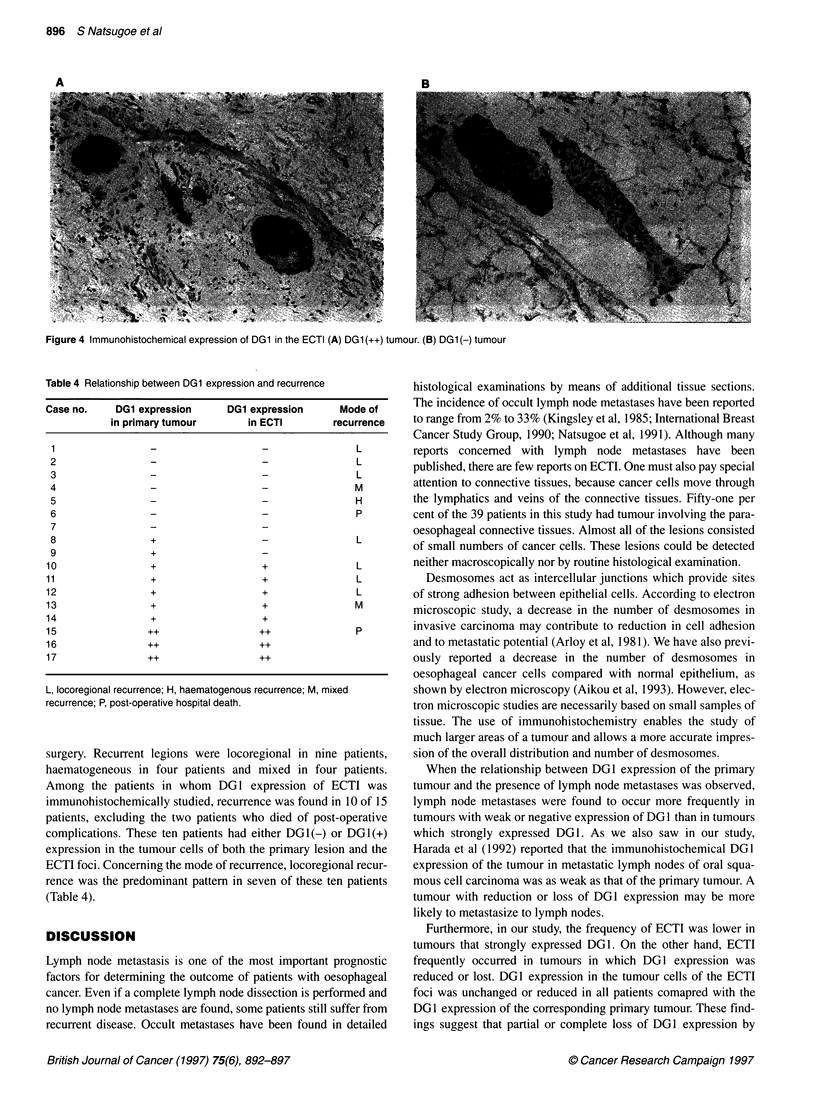

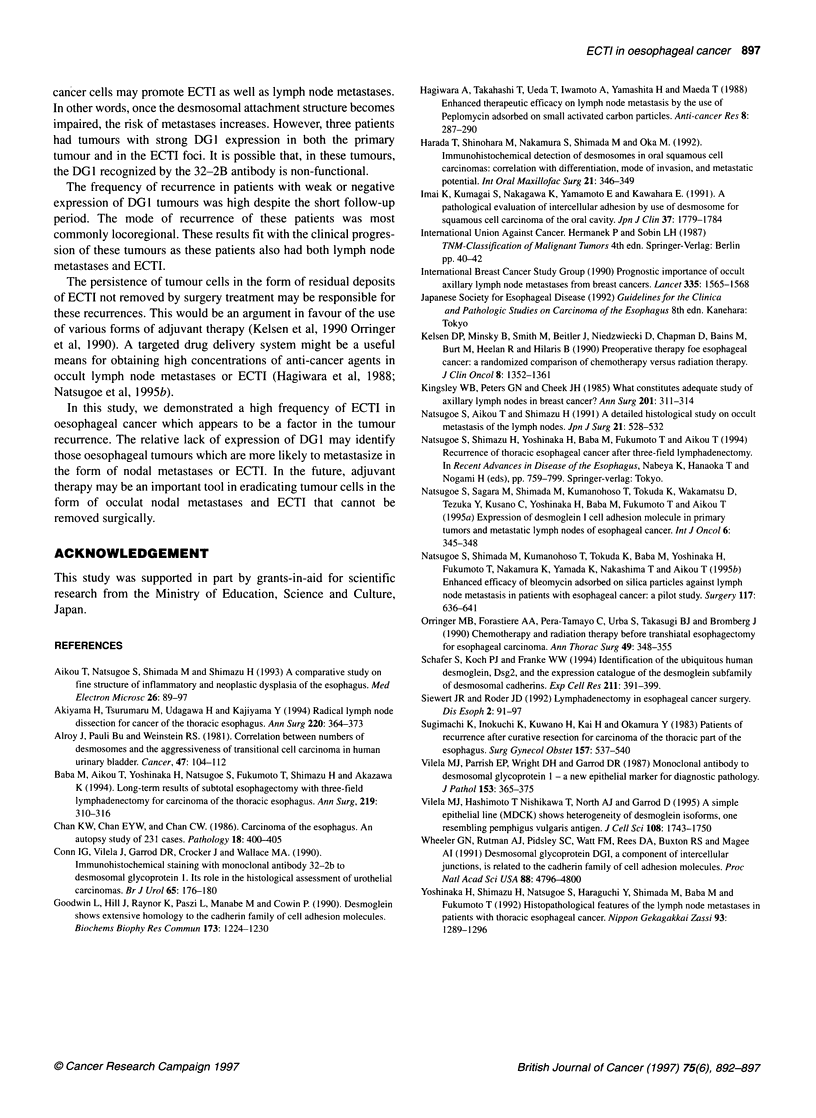


## References

[OCR_00510] Akiyama H., Tsurumaru M., Udagawa H., Kajiyama Y. (1994). Radical lymph node dissection for cancer of the thoracic esophagus.. Ann Surg.

[OCR_00513] Alroy J., Pauli B. U., Weinstein R. S. (1981). Correlation between numbers of desmosomes and the aggressiveness of transitional cell carcinoma in human urinary bladder.. Cancer.

[OCR_00520] Baba M., Aikou T., Yoshinaka H., Natsugoe S., Fukumoto T., Shimazu H., Akazawa K. (1994). Long-term results of subtotal esophagectomy with three-field lymphadenectomy for carcinoma of the thoracic esophagus.. Ann Surg.

[OCR_00525] Chan K. W., Chan E. Y., Chan C. W. (1986). Carcinoma of the esophagus. An autopsy study of 231 cases.. Pathology.

[OCR_00529] Conn I. G., Vilela M. J., Garrod D. R., Crocker J., Wallace D. M. (1990). Immunohistochemical staining with monoclonal antibody 32-2B to desmosomal glycoprotein 1. Its role in the histological assessment of urothelial carcinomas.. Br J Urol.

[OCR_00536] Goodwin L., Hill J. E., Raynor K., Raszi L., Manabe M., Cowin P. (1990). Desmoglein shows extensive homology to the cadherin family of cell adhesion molecules.. Biochem Biophys Res Commun.

[OCR_00541] Hagiwara A., Takahashi T., Ueda T., Iwamoto A., Yamashita H., Maeda T. (1988). Enhanced therapeutic efficacy on lymph node metastasis by the use of peplomycin adsorbed on small activated carbon particles.. Anticancer Res.

[OCR_00548] Harada T., Shinohara M., Nakamura S., Shimada M., Oka M. (1992). Immunohistochemical detection of desmosomes in oral squamous cell carcinomas: correlation with differentiation, mode of invasion, and metastatic potential.. Int J Oral Maxillofac Surg.

[OCR_00570] Kelsen D. P., Minsky B., Smith M., Beitler J., Niedzwiecki D., Chapman D., Bains M., Burt M., Heelan R., Hilaris B. (1990). Preoperative therapy for esophageal cancer: a randomized comparison of chemotherapy versus radiation therapy.. J Clin Oncol.

[OCR_00578] Kingsley W. B., Peters G. N., Cheek J. H. (1985). What constitutes adequate study of axillary lymph nodes in breast cancer?. Ann Surg.

[OCR_00582] Natsugoe S., Aiko T., Shimazu H. (1991). A detailed histological study on occult metastasis of the lymph nodes.. Jpn J Surg.

[OCR_00600] Natsugoe S., Shimada M., Kumanohoso T., Tokuda K., Baba M., Yoshinaka H., Fukumoto T., Nakamura K., Yamada K., Nakashima T. (1995). Enhanced efficacy of bleomycin adsorbed on silica particles against lymph node metastasis in patients with esophageal cancer: a pilot study.. Surgery.

[OCR_00608] Orringer M. B., Forastiere A. A., Perez-Tamayo C., Urba S., Takasugi B. J., Bromberg J. (1990). Chemotherapy and radiation therapy before transhiatal esophagectomy for esophageal carcinoma.. Ann Thorac Surg.

[OCR_00613] Schäfer S., Koch P. J., Franke W. W. (1994). Identification of the ubiquitous human desmoglein, Dsg2, and the expression catalogue of the desmoglein subfamily of desmosomal cadherins.. Exp Cell Res.

[OCR_00622] Sugimachi K., Inokuchi K., Kuwano H., Kai H., Okamura T., Okudaira Y. (1983). Patterns of recurrence after curative resection for carcinoma of the thoracic part of the esophagus.. Surg Gynecol Obstet.

[OCR_00632] Vilela M. J., Hashimoto T., Nishikawa T., North A. J., Garrod D. (1995). A simple epithelial cell line (MDCK) shows heterogeneity of desmoglein isoforms, one resembling pemphigus vulgaris antigen.. J Cell Sci.

[OCR_00627] Vilela M. J., Parrish E. P., Wright D. H., Garrod D. R. (1987). Monoclonal antibody to desmosomal glycoprotein 1--a new epithelial marker for diagnostic pathology.. J Pathol.

[OCR_00637] Wheeler G. N., Parker A. E., Thomas C. L., Ataliotis P., Poynter D., Arnemann J., Rutman A. J., Pidsley S. C., Watt F. M., Rees D. A. (1991). Desmosomal glycoprotein DGI, a component of intercellular desmosome junctions, is related to the cadherin family of cell adhesion molecules.. Proc Natl Acad Sci U S A.

[OCR_00644] Yoshinaka H., Shimazu H., Natsugoe S., Haraguchi Y., Shimada M., Baba M., Fukumoto T. (1992). [Histopathological features of the lymph node metastases in patients with thoracic esophageal cancer].. Nihon Geka Gakkai Zasshi.

